# Generation of FGF reporter transgenic zebrafish and their utility in chemical screens

**DOI:** 10.1186/1471-213X-7-62

**Published:** 2007-06-06

**Authors:** Gabriela A Molina, Simon C Watkins, Michael Tsang

**Affiliations:** 1 Department of Molecular Genetics and Biochemistry, University of Pittsburgh, School of Medicine. BST3-5062, 3051 Fifth Avenue, Pittsburgh, PA 15213, USA; 2 Department of Cell Biology and Physiology, Center for Biological Imaging, University of Pittsburgh, School of Medicine S362 Biomedical Science Towers, 3500 Terrace Street. Pittsburgh, PA 15261 USA; 3 LMG/NICHD/NIH, Building 6B, 9000 Rockville Pike. Bethesda, MD 20892 USA

## Abstract

**Background:**

Fibroblast Growth Factors (FGFs) represent a large family of secreted proteins that are required for proper development and physiological processes. Mutations in mouse and zebrafish FGFs result in abnormal embryogenesis and lethality. A key to understanding the precise role for these factors is to determine their spatial and temporal activity during embryogenesis.

**Results:**

Expression of *Dual Specificity Phosphatase 6 *(*dusp6*, also known as *Mkp3*) is controlled by FGF signalling throughout development. The *Dusp6 *promoter was isolated from zebrafish and used to drive expression of destabilized green fluorescent protein (*d2EGFP*) in transgenic embryos (*Tg(Dusp6:d2EGFP)*). Expression of d2EGFP is initiated as early as 4 hours post-fertilization (hpf) within the future dorsal region of the embryo, where *fgf3 *and *fgf8 *are initially expressed. At later stages, d2EGFP is detected within structures that correlate with the expression of *Fgf *ligands and their receptors. This includes the mid-hindbrain boundary (MHB), pharyngeal endoderm, otic vesicle, hindbrain, and Kupffer's vesicle. The expression of d2EGFP is under the control of FGF signalling as treatment with FGF Receptor (FGFR) inhibitors results in the suppression of d2EGFP expression. In a pilot screen of commercially available small molecules we have evaluated the effectiveness of the transgenic lines to identify specific FGF inhibitors within the class of indolinones. These compounds were counter screened with the transgenic line *Tg(Fli1:EGFP)*^*y*1^, that serves as an indirect read-out for Vascular Endothelial Growth Factor (VEGF) signalling in order to determine the specificity between related receptor tyrosine kinases (RTKs). From these assays it is possible to determine the specificity of these indolinones towards specific RTK signalling pathways. This has enabled the identification of compounds that can block specifically the VEGFR or the FGFR signalling pathway.

**Conclusion:**

The generation of transgenic reporter zebrafish lines has allowed direct visualization of FGF signalling within the developing embryo. These FGF reporter transgenic lines provide a tool to screen for specific compounds that can distinguish between two conserved members of the RTK family.

## Background

The complex process of embryogenesis is directed by the regulation of signalling pathways that are achieved in part by the activity of a variety of secreted ligands. Thus understanding the temporal and spatial activity of signalling peptides is key to determining the role for these factors in controlling cellular fates. For example, Fibroblast Growth Factors (FGFs), a family of secreted glycoproteins, perform crucial functions that include the establishment of embryo polarity, the formation of organizing centres, and the induction of limb outgrowth [1-3]. These ligands are expressed in discrete domains during development and their actions are restricted to cells that express integral membrane proteins that can bind FGFs [2,4]. The FGF receptors (FGFRs) are members of the receptor tyrosine kinase (RTK) class of transmembrane proteins and activate several signalling cascades, including the phospholipase C gamma (PLC-γ), phosphatidylinositol-3 kinase (PI3K) which activates Akt/protein kinase B, and Ras which activates extracellular signal-regulated protein kinase (ERK, also known as MAPK) pathways [5,6]. FGF activity results in the control of gene expression through the modification of transcription factors by activated ERKs and AKT. As a consequence of altered gene expression, cellular proliferation, survival and fate determination can be governed by FGF activity. How FGFs control gene expression and the nature of the genes that they regulate during development is still not completely established.

One step towards defining FGF target genes is to determine the temporal and spatial activity of FGFs during development. This will provide an activity map of where and when these factors act to control developmental processes. Since activation of FGF signalling results in the phosphorylation of Erk, one approach to illustrate FGF activity during development has been to detect the spatial and temporal presence of phosphorylated ERKs in the embryo. This has resulted in mapping the location of FGF activity during mouse, chick, *Xenopus laevis *and zebrafish embryogenesis [7-12]. While these studies provide a detailed analysis of FGF activity during development, it is not possible to visualize FGF activity in the live embryo and observe the dynamic changes in FGF signalling as the embryo develops.

We have previously identified several FGF regulated genes in zebrafish, including the *Dual Specificity Phosphatase 6, dusp6 (also known as Map Kinase Phosphatase 3, mkp3)*, and *Sef*, two genes that exhibit almost identical expression to *fgf8 *and *fgf3 *during development [11,13-15]. Dusp6 functions to dephosphorylate activated p44 and p42 ERKs and over-expression of Dusp6 results in the suppression of FGF activity in the embryo [11,16-18]. Expression of *dusp6 *was suppressed in embryos treated with SU5402, an FGFR inhibitor, or by the ectopic expression of dominant negative FGFR, indicating that *dusp6 *transcription is regulated by FGFs [11,16-18]. Genetic studies in mouse have identified the requirement for FGFRs in maintaining *Dusp6 *expression, as loss of either FGFR1 or FGFR2 resulted in the depletion of *Dusp6 *transcripts [19]. It is clear from these studies that *Dusp6 *expression is regulated by FGF ligands and receptors, however it has been controversial as to which signalling pathway downstream of the receptor is required for *Dusp6 *gene transcription.

Experiments described in the chick, mouse and zebrafish embryos have provided clues that *Dusp6 *gene regulation is context dependent [11,16-18,20,21]. In several studies, the PI3K inhibitor, LY294002, was used in the chick limb bud to show that blocking the PI3K/AKT pathway results in the suppression of *Dusp6 *expression within the distal limb bud [17,20]. Likewise, implantation beads soaked in LY294002 could suppress *Dusp6 *expression within the mid-hindbrain boundary (MHB) in mouse and chick embryos [18,21]. In contrast, genetic studies in the mouse has revealed that the PI3K pathway is not required as knock-out of PDK1, an upstream activator of PI3K, still allowed expression of *Dusp6 *albeit in a disorganized fashion [20].

Analysis of the RAS/MAPK pathway under similar experimental conditions indicates that this pathway is also important for *Dusp6 *gene expression. For instance, the implantation of beads soaked with PD184352, a specific inhibitor of Mek can also block *Dusp6 *expression in the chick limb bud and somites, implicating the RAS/MAPK pathway in regulating *Dusp6 *expression [16,20,22]. Further, over-expression of Dusp6 itself, which would result in the removal of activated ERKs and hence the shutdown the RAS/MAPK pathway, resulted in the down regulation of *Dusp6 *transcription [11,20]. Although these studies highlight the complex nature of *Dusp6 *gene regulation downstream of the FGF receptor it is however clear that *Dusp6 *is a direct target of FGF signalling. This concept is supported by the fact that the presence of Erk phosphorylation correlates with *Dusp6 *expression throughout chick embryogenesis [12]. A detailed map of ERK activation was described and *Dusp6 *expression was found to colocalize with activated ERKs in all tissues throughout chick development [12]. Thus FGF activity can be directly measured by either the presence of phosphorylated ERKs or indirectly by the presence of *Dusp6 *transcripts.

The generation of transgenic lines that express fluorescent reporters in response to FGFs would allow live visualization of FGF activity during development. Since zebrafish embryos develop *ex utero*, the direct visualization of d2EGFP in the embryos can provide an indirect biosensor for FGF activity *in vivo*. In this report we have generated transgenic zebrafish lines that expresses a destabilized form of Green Fluorescent Protein (d2EGFP) under the control of active FGF signalling. To achieve this we isolated the promoter region of the *dusp6 *gene and fused it to *d2EGFP *reporter gene. d2EGFP fluorescence can be visualised in transgenic embryos within multiple tissues where activated ERKs (phosphorylated) have been detected, and where expression of FGF ligands, receptors and target genes such as *sef*, *sprouty4, pea3 *and *erm *have been described [9,10,13,15,23-31].

The zebrafish has become a viable model organism for chemical screens [32-34]. With the generation of fluorescent transgenic reporter zebrafish lines it is possible to discover specific molecules that can alter differentiation events during organogenesis [35]. We have validated the FGF reporter lines as tools to identify novel FGF modulators and in counter screens it is possible to determine compound specificity towards two closely related RTK pathways. The generation of *in vivo *reporters for FGF activity will provide a valuable tool to screen for genes or chemicals that modulate FGF signalling.

## Results

### Generation of transgenic FGF reporter lines

**Figure 1:**
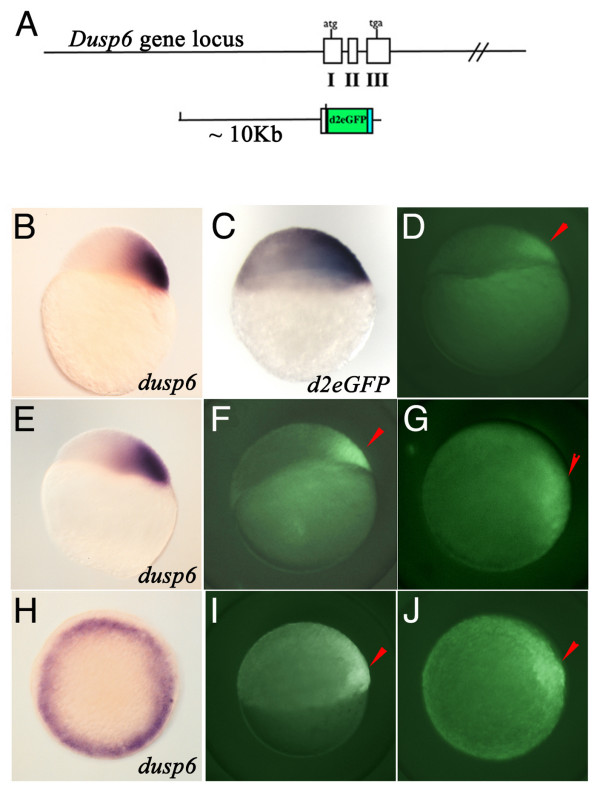
**Generation of *Dusp6 *DNA construct and expression of d2EGFP in transgenic embryos**. **(A) **Diagram showing the *Dusp6 *gene locus and the DNA construct used in generating transgenic zebrafish. **(B, E, & H) ***dusp6 *expression at oblong **(B)**, dome **(E)**, and shield stage. **(C) ***d2EGFP mRNA *expression at sphere stage. **(D, F, G, I, & J) ***Tg(Dusp6:d2EGFP)*^*pt*6 ^embryos at dome **(D)**, 30% epiboly **(F & G)**, and shield **(I & J) **stage. **(B-F & I) **are lateral views and **(H & J) **are animal views. Red arrowheads mark dorsal region of the embryo.

The zebrafish *Dusp6 *gene locus was identified by PCR screening a BAC library from Genome Systems. The BAC clone contained the full gene *Dusp6 *locus, and a 10 Kb fragment that includes the 5' untranslated sequence within exon I was subcloned into a vector containing the *d2EGFP*, a gene that encodes a destabilised green fluorescent protein that has a two hour half-life (Figure 1A) [36]. Since FGF signalling during embryogenesis is dynamic, we reasoned that a destabilized form of EGFP would likely recapitulate the endogenous expression of *dusp6 *mRNA. 1-cell stage zebrafish embryos were co-injected with the transgenic DNA construct and I-Sce 1 meganuclease as described by Thermes *et al*. to generate transgenic lines [37]. Injected embryos were raised to adulthood and screened for potential germline carriers by d2EGFP expression. Ten founder transgenic animals were identified in the F1 generation. While d2EGFP expression levels differed between each of the transgenic lines, the expression patterns were identical between the transgenic lines. We maintained four of the strongest lines (*Tg(Dusp6:d2EGFP)*^*pt*6-*pt*9^*)*, and for this study we utilized the line *Tg(Dusp6:d2EGFP)*^*pt*6^. We have generated homozygous lines for several of the transgenes and observed the expected Mendelian inheritance. Further, there were no deleterious effects noted in transgenic animals, suggesting that insertion of the DNA construct did not impact zebrafish growth and development.

### Expression of d2EGFP is localized to FGF signalling centres in transgenic embryos

d2EGFP was first visualized as early as dome stage within the prospective dorsal region of the embryo, consistent with the restricted dorsal expression of *dusp6 mRNA *and with the initial expression patterns of *fgf3 *and *-8 *(compare Figure 1B to 1D) [11]. The fluorescent pattern was supported by the expression of *d2EGFP mRNA *as tested by *in situ *hybridisation (Figure 1C). At sphere stage, *d2EGFP *expression is maintained within the dorsal region and colocalises with *dusp6 *expression, a domain where the organiser (shield) eventually forms (compare Figure 1E to 1F). At 6 hpf, the highest d2EGFP expression is detected within the shield, and weakly throughout the margin, which resembles the pattern of *dusp6 mRNA *at this stage (compare Figure 1J to 1H).

**Figure 2:**
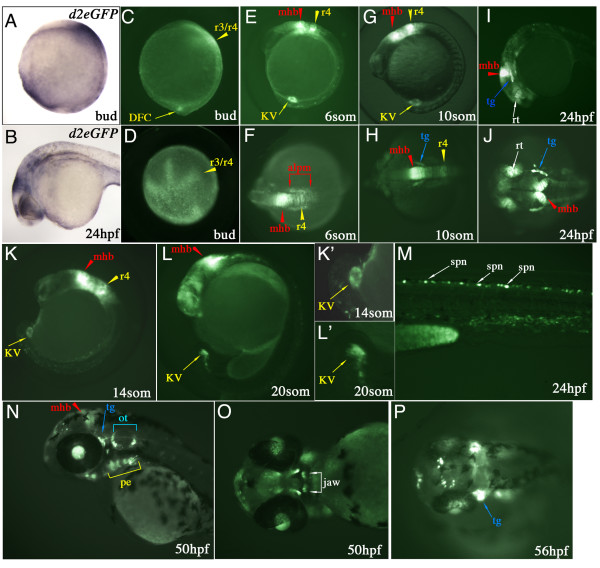
**Spatial and temporal d2EGFP expression in *Tg(Dusp6:d2EGFP)*^*pt*6 ^embryos**. **(A & B) **Lateral views of *d2EGFP mRNA *expression at Bud stage and 24 hpf. **(C-P) **d2EGFP expression in *Tg(Dusp6:d2EGFP)*^*pt*6 ^embryos, stages are indicated in each panel. At bud stage **(C & D)**, d2EGFP is detected in the hindbrain (r3/r4, yellow arrowhead) and within the caudal region in the DFCs. **(E, G & K) **From 8- to 14-somite stages, lateral views show expression of d2EGFP in cells lining Kupffer's vesicle, within r4 (r4, yellow arrowhead) and the mid-hindbrain boundary (mhb, red arrowhead). **(F & H) **Dorsal views show high d2EGFP expression within the MHB, r4 and the anterior lateral plate mesoderm (alpm, red brackets). **(H) **At 10-somite stage initial d2EGFP expression is detected within the trigeminal ganglia (tg, blue arrow). **(I & J) **24 hpf embryo showing d2EGFP expression in the MHB, trigeminal ganglia, dorsal retina (rt, white arrow) and pharyngeal endoderm (pe, yellow bracket). **(K & L) **14 and 20-somite stage embryo highlighting the expression of d2EGFP in Kupffer's vesicle. Higher magnifications are show in **(K' & L')**. **(M) **Trunk region shows d2EGFP expression within the dorsal spinal cord neurons (spn, white arrow) at 24 hpf. **(N) **At 50 hpf expression is noted in the MHB, trigeminal ganglia, pharyngeal endoderm and otic vesicle (ot, blue bracket). **(O) **Ventral view of 50 hpf, showing d2EGFP expression in the jaw (white bracket). **(P) **At 56 hpf, strong expression in noted in the trigeminal ganglia, the jaw and also in neurons within the dorsal diencephalon.

Reporter gene expression was also analysed at later stages by *in situ *hybridisation performed to detect the presence of *d2EGFP mRNA*. At bud stage (10 hpf), *d2EGFP *transcripts can be detected within the posterior ventral domain and within the presumptive hindbrain, where *fgf8 *expression has been noted (Figure 2A) [14,30,31]. By 24 hpf, *d2EGFP mRNA *is detected within the MHB, pharyngeal arches, otic vesicle, retina, optic stalk and dorsal diencephalon (Figure 2B). The ligands *fgf8*, *fgf17 *and *fgf3*, and FGFRs are known to be expressed within these same domains, suggesting that *mRNA *expression of this reporter gene is under the control of FGF signalling [14,23,25,26,30,31]. In comparison to the fluorescent protein expression, *d2EGFP *transcripts can be detected in a much wider domain and also more prominent (compare Figure 2A to 2C and Figure 2B to 2I). One reason for the discrepancy can be attributed to the threshold level required for visualisation of d2EGFP protein as compared to the detection of transcripts by *in situ *hybridisation.

At bud stage, fluorescent protein can be visualized within the medial neural plate, presumptive hindbrain and at a position that corresponds to the site where the Dorsal Forerunner Cells (DFCs) in the posterior domain of transgenic embryos have been described (Figure 2C &2D) [38,39]. The DFCs represent a specialized group of non-involuting cells that migrate just ahead of the shield during gastrulation and eventually forms a fluid filled structure known as Kupffer's vesicle, a transient organ particular to teleosts [38,39]. Kupffer's vesicle is thought to serve equivalent functions in establishing left-right polarity as the mouse node [40-43].

By the 6-somite stage, strong d2EGFP fluorescence can be detected in the MHB, the hindbrain with strongest expression in r4, the anterior lateral plate mesoderm and the Kupffer's vesicle (Figure 2E &2F). Approximately two hours later at the 10-somite stage expression of d2EGFP is located within the same domains as described for the 6-somite stage with the addition of the trigeminal ganglia (Figure 2G &2H). The expression within Kupffer's vesicle is quite striking as the structure is completely outlined by d2EGFP positive cells from the 6-somite onwards (Figure 2C, 2E, 2G, 2K and 2K'). In the zebrafish, a role for FGF signalling has been suggested in the formation of Kupffer's vesicle, as *fgf8 *is expressed in the DFCs at gastrulation, and this structure is absent in about 30% of *ace(fgf8) *mutants [44]. The *Tg(Dusp6:d2EGFP)*^*pt*6 ^line confirms that the DFCs and Kupffer's vesicle receive FGF signals from the time when the DFCs begin to coalesce and right through to the 20-somite stage when Kupffer's vesicle begins to collapse (Figure 2K, 2L, 2K', &2L'). Time lapse imaging of the transgenic embryos shows fluorescent DFCs migration towards the posterior region of the embryo and the formation of Kupffer's Vesicle at the 6-somite stage [see Additional files 1, 2, 3 and 4]. d2EGFP expression is still noted throughout these stages and even after the collapse of Kupffer's vescles when these epithelial cells migrate towards the tail bud and contribute to mesodermal tissues such as notochord, posterior somites and the tail bud (see Additional files 2, 3 and 4) [38,39]. By the 26-somite stage, the Kupffer's vesicle cells have lost their expression of d2EGFP, suggesting that these cells no longer receive FGF signals (data not shown).

At 24 hpf, d2EGFP can be visualized within the dorsal retina, trigeminal ganglia, otic vesicles, within the dorsal diencephalon, and dorsal spinal cord neurons (Figure 2I, 2J &2M). These domains of d2EGFP expression are consistent with expression of FGF ligands, receptors and known target genes such as *erm*, *pea3*, *sef *and *sprouty4*, suggesting that the transgenic line reports on FGF activity *in vivo *[13,15,24,27-29]. d2EGFP expression persists throughout the next day of development in a majority of the same domains as at 24 hpf and continues up to 56 hpf (Figure 2N, 2O &2P). The one exception is that dorsal retina expression is lost, while lens expression becomes stronger from 36 hpf onwards (Figure 2N). Expression at later stages can also be detected within the developing jaw and further refined within the pharyngeal arches and the pectoral fins (Figure 2N &2O, pectoral fins not shown). A dorsal view of a 56 hpf transgenic embryo reveals d2EGFP expression within distinct cells in the dorsal diencephalon, suggesting that these particular cells are responding to FGF signals (Figure 2P). Of particular interest is that there seems to be an asymmetric distribution of fluorescent cells with respect to the left-right axis, implying that within this region of the diencephalon, FGF signalling is asymmetrical (Figure 2P). An alternative view is that the asymmetric expression of d2EGFP is a result of anatomical or stochastic (random) differences between the left and right side of the diencephalon. Studies have shown that the left habenular nuclei is larger and the parapineal gland is situated to the left of the midline, suggesting that d2EGFP expression reflects structural difference between the left and right dorsal diencephalon [45,46].

The domains of d2EGFP expression in the transgenic embryos were consistent with many of the regions where endogenous *dusp6 *transcripts have been detected. However, *dusp6 mRNA *expression was not fully recapitulated in the transgenic embryos, in particular FGF activity and *dusp6 *expression have been well documented within the somites and tail bud and yet no expression of *d2EGFP mRNA *or protein were detected in these regions (Figure 2) [11]. One explanation is that only 10 Kb of upstream *Dusp6 *promoter sequence was used to generate the DNA construct for transgenesis, hence it is likely that somitic and tail bud enhancers were not present within this sequence.

In summary we have generated several transgenic lines that express d2EGFP under the control of the *Dusp6 *promoter, a gene that is directly regulated by FGF signalling during development. Since d2EGFP has a half life of just two hours, it is likely that domains of high d2EGFP expression represents cells that respond to active FGF signalling as opposed to cells inheriting fluorescent protein from their ancestors.

### FGF signalling controls reporter gene expression

**Figure 3:**
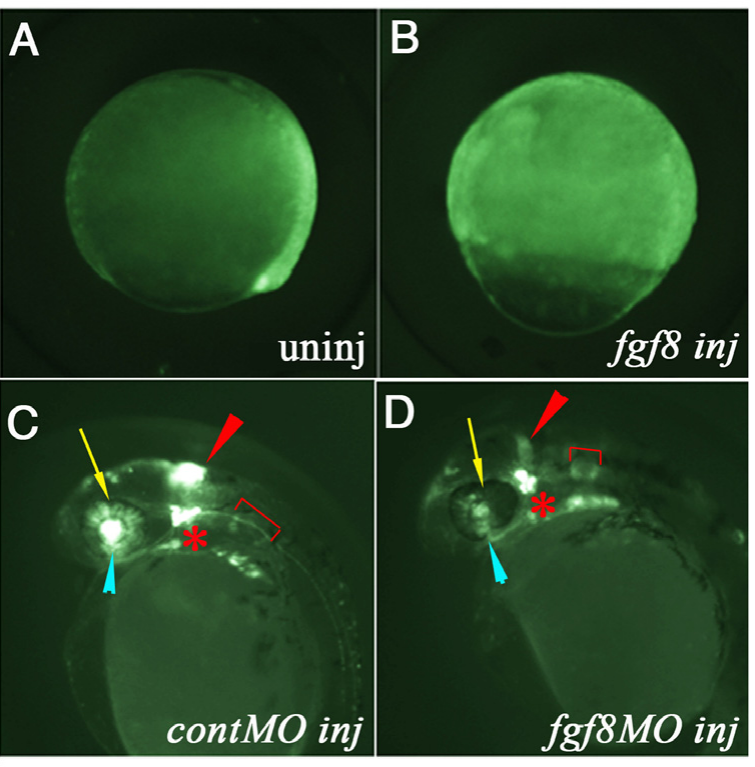
**D2EGFP expression is dependant on FGF signaling**. **(A & B) **Lateral views of gastrula staged *Tg(Dusp6:d2EGFP)*^*pt*6 ^embryos. Control uninjected is shown in **(A)**, while in **(B) ***fgf8 mRNA *injected at the 2-cell stage. Expression of d2EGFP is greatly expanded in *fgf8*-injected embryos. **(C & D) **Lateral views of 28 hpf transgenic embryos. **(C) **Control MO injected **(D) ***fgf8-MO *injected embryo shows loss of d2EGFP expression within the MHB, dorsal retina and smaller otic vesicles. Red arrowhead: MHB; yellow arrow: dorsal retinal; blue arrowhead: optic stalk; red bracket: otic vesicle.

**Figure 4:**
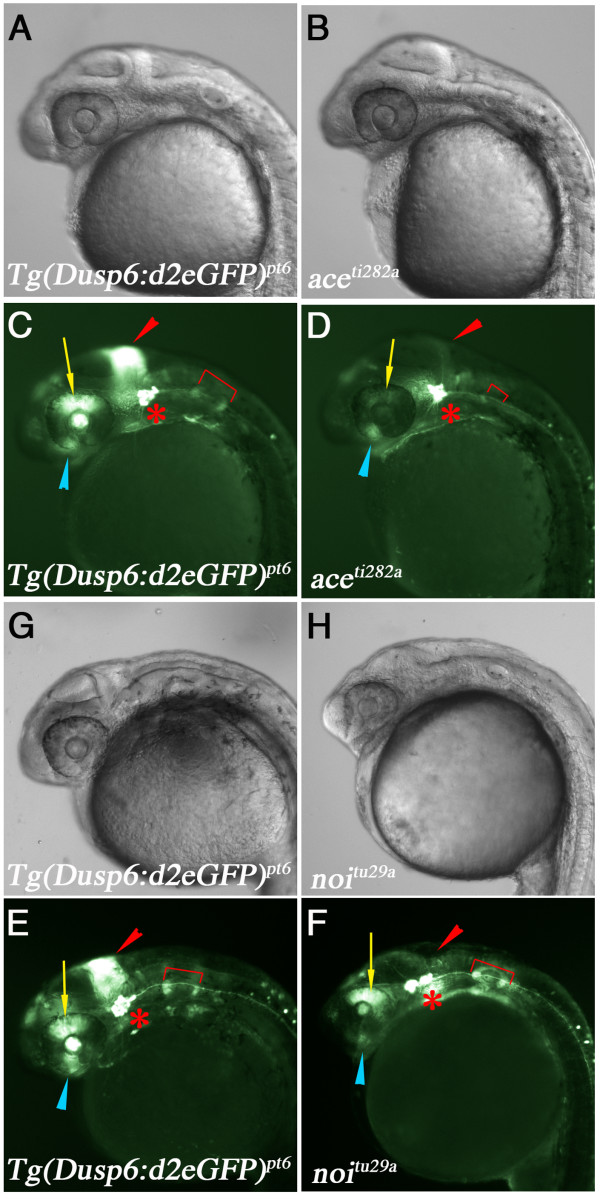
**D2EGFP expression in *fgf8/ace *and *pax2.1/noi *mutants**. **(A-F) **Lateral views of *Tg(dusp6:d2EGFP)*^*pt*6 ^embryos at 28 hpf crossed into *ace *or *noi *mutants. Genotype is listed in bottom left corner. **(A & B) **Brightfield images of wildtype sibling and *ace *mutant embryo, respectively. **(C & D) **d2EGFP expression in WT sibling and *ace *mutant. Note loss of d2EGFP expression within the MHB, dorsal retina, and the smaller otic vesicle, while d2EGFP expression in the trigeminal ganglia is unaffected. **(G & H) **Brightfield images of wildtype sibling and *noi *mutant embryo. **(E & F) **d2EGFP expression is lost in the MHB and optic stalk. In contrast to the *ace *mutants, expression within the dorsal retina and otic vesicles are normal.

To test whether d2EGFP expression is responsive to experimental modulation in FGF signalling, we injected *fgf8 *mRNA (10 pg) into 2–4 cell stage *Tg(Dusp6:d2EGFP)*^*pt*6 ^embryos. As expected, expression of d2EGFP was greatly expanded in *fgf8*-injected embryos at the gastrula stage (compare Figure 3B to 3A). Conversely, injection of *fgf8*-MOs (10 ng) resulted in a suppression of d2EGFP expression within the MHB and retina, while d2EGFP expression within the trigeminal ganglia was unaffected (compare Figure 3D to 3C). To confirm these findings we analyzed d2EGFP expression in two mutant zebrafish lines that exhibit defective FGF signalling [31,47]. We in-crossed *Tg(Dusp6:d2EGFP)*^*pt*6 ^into the *ace(fgf8) *mutant and expression of d2EGFP within the MHB was greatly diminished in mutant embryos from 20 hpf onwards (not shown). This is clearly evident at 28 hpf when the MHB is morphologically distinguishable between wildtype siblings and mutants (Figure 4A–D). Furthermore expression within the retina and lens was absent, while expression in the optic stalk and trigeminal ganglia were unaffected at 28 hpf (Figure 4A–D). In the *noi(pax2.1) *mutant, d2EGFP expression was markedly reduced in the MHB, where FGF signalling is absent, however expression within the retina, lens, trigeminal ganglia and otic vesicles was unaltered. In contrast to the *ace *mutants, expression of d2EGFP within the optic stalk is severely reduced as this structure fails to from in the *noi *mutants (compare Figure 4E to 4F) Taken together, these results show that we have generated a transgenic reporter line that expresses d2EGFP under the control of FGF signalling during development.

### Treatment of *Tg(Dusp6:d2EGFP)*^*pt*6 ^embryos with known FGF pathway inhibitors

Given that *Tg(Dusp6:d2EGFP)*^*pt*6 ^embryos respond to changes in FGF signalling, these embryos can provide a valuable tool to screen for molecules that modulate FGF signalling *in vivo*. To validate that these reporter lines can measure changes in FGF activity in a chemical screen, *Tg(Dusp6:d2EGFP)*^*pt*6 ^embryos were treated with known inhibitors of the FGF pathway. For our studies we selected transgenic embryos at 24 hpf and treated with compounds for 8 hours. We reasoned that since the half-life of d2EGFP is 2 hours, then an inhibitor of FGF signalling should substantially reduce d2EGFP fluorescence after 6–8 hours of treatment. Furthermore, since embryos at this developmental stage (24 hpf) have already formed many of the structures that express d2EGFP such as the MHB, trigeminal ganglia and otic vesicles, then the assay would directly measure loss of d2EGFP fluorescence as a consequence of FGF inhibition and not per se a loss of these tissues.

**Figure 5:**
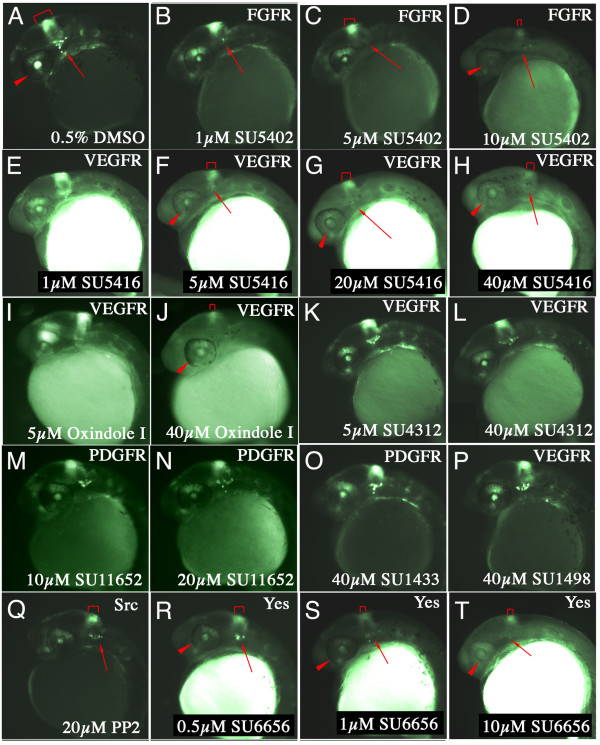
**Validation of *Tg(Dusp6:d2EGFP)*^*pt*6 ^line for chemical screening**. **(A-Y) **Lateral views of 30 hpf embryos treated with the compound and dose are indicated on the bottom right. The protein targets for these compounds are listed in the top right hand corner. **(A) **Embryo incubated in 0.5% DMSO as control. **(B-D) **Increasing doses of SU5402 suppressed d2EGFP expression in the MHB (red bracket), trigeminal ganglia (red arrow) and dorsal retina (red arrowhead). **(E-H) **Increasing doses of SU5416, a non-specific inhibitor of VEGFRs, suppressed FGF signalling. **(I & J) **Oxindole I another related VEGFR inhibitor also suppressed d2EGFP fluorescence in transgenic embryos. **(K-N) **In contrast, two compounds with similar chemical structure SU4312 and SU11652, did not block FGF signalling. **(O & P) **Likewise, two unrelated inhibitors of PDGFR and VEGFR, SU1433 and SU1498 also failed to suppress d2EGFP expression. **(Q-T) **PP2 and SU6656, two Src Kinase inhibitors suppressed FGF signalling in transgenic embryos.

We treated transgenic embryos with the indolinone FGF receptor inhibitor, SU5402 [Additional file 5 shows the structure of the compounds used in this study] [48]. SU5402 has been extensively used to block FGF signalling in zebrafish and *Xenopus laevis *embryos with doses ranging from 20 μM to 160 μM [10,27,49,50]. In this study, 1 μM SU5402 was effective at suppressing expression of d2EGFP within the trigeminal ganglia and reducing MHB fluorescence after only 8 hours of treatment (compare Figure 5A to 5B). At 5 μM of SU5402, expression of d2EGFP was markedly reduced within the MHB, and even eliminated within the dorsal retina and trigeminal ganglia (Figure 5C). At 10 μM, d2EGFP fluorescence in the transgenic embryos was completely abolished (Figure 5D).

Treatment with other receptor tyrosine kinase inhibitors was also performed to determine the specificity of this assay for identifying other small molecules that can modulate FGF signalling. We assessed the activity of several Vascular Endothelial Growth Factor Receptor (VEGFR) and Platelet Derived Growth Factor Receptor (PDGFR) inhibitors to reveal if these compounds, structurally related to SU5402, could suppress d2EGFP expression in the FGF reporter lines. We reasoned that most of these compounds contain the same indolinone backbone as SU5402, and therefore some would also block FGFR signalling in the zebrafish. Thus the analysis of these compounds in the FGF reporter line can determine the chemical structures that suppress FGF signalling from a family of related molecules. One of the first indolinones to undergo clinical trials was SU5416, a reported specific inhibitor for VEGFR2 [see Additional file 5] [51-53]. SU5416 was added to 24 hpf stage transgenic embryos for 6 hours to determine if this compound is specific for VEGFR versus FGFR. While this compound exhibits autofluorescence, especially within the yolk, the expression of d2EGFP did not markedly change at 1 μM (Figure 5E). In contrast at 5 μM, the trigeminal ganglia and MHB fluorescence was markedly diminished (Figure 5F). At even higher doses, SU5416 completely eliminated d2EGFP expression within the transgenic embryos, even though the treated embryos exhibited strong fluorescent yolks, the expression of d2EGFP within the MHB was clearly eliminated (Figure 5G &5H). These results suggest that SU5416 is not completely specific to VEGFR2 at these concentrations and it can block FGF signalling. Similarly, Oxindole I was also effective at blocking d2EGFP expression, however a much higher dose (40 μM) was required [see Additional file 5], and (Figure 5I &5J). SU4312 represents another indolinone inhibitor of VEGFR, which has been shown to block autophosphorylation of VEGFRs [54]. In contrast to SU5416, treatment up to 40 μM of SU4312 had little effect in the expression of d2EGFP within the MHB and trigeminal ganglia in the *Tg(Dusp6:d2EGFP)*^*pt*6 ^embryos [see Additional file 5] and (Figure 5K &5L). A newer generation of indolinone compound was also tested to determine its specificity towards VEGFR/PDGFR versus FGFR. SU11652, has been shown to have at least 100-fold greater inhibitory effect targeting a VEGFR and PDGFR versus FGFR [see Additional file 5] [55,56]. In our assays, SU11652 did not alter d2EGFP expression suggesting that this compound does not block FGF signalling (Figure 5M &5N). We also treated transgenic embryos with two structurally unrelated VEGF signalling inhibitors, SU1498 and SU1433 (also known as AG1433) to determine the specificity of these molecules [see Additional file 5] [57-59]. Both compounds have been shown to be effective at blocking VEGFR, and to a lesser extent basic FGF in HUVEC tubulogenesis assays [58]. Treatment with high doses of SU1433 or SU1498 did not alter d2EGFP fluorescence in the transgenic embryos, suggesting that these chemically divergent compounds do not block FGF signalling in the zebrafish at the doses indicated (Figure 5O &5P).

We next determined the activity of Src Kinase inhibitors and their role in FGF signalling in the early embryo as previous reports have shown that several Src family members function to relay FGF signalling [60,61]. PP2, an inhibitor of several Src Kinases including Fyn and Lck as well as PDGFRs and Bcr-Abl, only mildly altered d2EGFP expression at 20 μM (Figure 5Q), a dose that is several fold higher than what has been shown to inhibit Fyn and Lck in cell culture [62,63]. SU6656, a more specific inhibitor of Src kinases, exhibited robust inhibitory activity in these assays. At concentrations of 1 μM, expression of d2EGFP was completely abolished within the MHB, trigeminal ganglia and the retina. The concentration of SU6656 was within the range that is known to be specific for blocking mouse Src kinase activity and not other related tyrosine kinases (Figure 5R–T) [63]. In fact 1 μM SU6656 is likely to inhibit only the Src members, Yes, Fyn, Src and Lyn, with Yes kinase exhibiting higher sensitivity, suggesting that Yes is required for FGF signalling in the zebrafish embryo [63].

The results from these experiments support the use of transgenic FGF reporter lines to screen for small molecules that affect FGF signalling. Given the rapid nature of these screens as d2EGFP expression can be suppressed in just 6 hours, it is likely that a chemical screen would identify compounds that directly modulate FGF signalling.

### Suppression of *dusp6 *expression in embryos treated with FGF inhibitors

**Figure 6:**
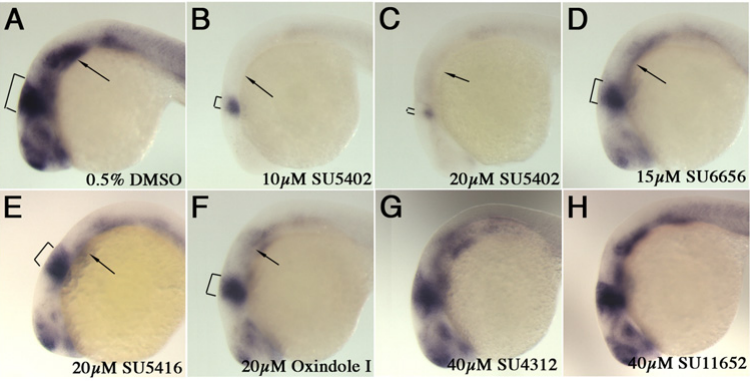
**Expression of *dusp6 *in chemically treated embryos**. **(A-H) **Lateral views of 24 hpf embryos treated with compounds indicated on bottom left and probed for the presence of *dusp6 *transcripts. **(A) **DMSO control, *dusp6 *is strongly expressed in the MHB (brackets), pharyngeal endoderm (arrow). **(B) **SU5402 at 10 μM greatly suppresses dusp6 transcription, while at a higher dose, 20 μM **(C) ***dusp6 *expression is almost eliminated. **(D-F) **SU6656, SU5416 and Oxindole I treated embryos exhibited weaker *dusp6 *expression. **(G & H) **SU4312 and SU11652 did not significantly alter *dusp6 *expression.

To confirm that suppression of d2EGFP fluorescence reflects reduced FGF target gene transcription, we analyzed *dusp6 *mRNA expression in wildtype embryos treated with chemical inhibitors (Figure 6). As predicted, compounds that suppressed fluorescence in *Tg(Dusp6:d2EGFP*)^*pt*6^embryos also resulted in a reduction of *dusp6 *transcripts (Figure 6B to 6F). SU5402, SU6656, SU5416 and Oxindole I all suppressed *dusp6 *expression after 10hr treatment at the dose indicated. In particular, *dusp6 *expression within the dorsal diencephalon, pharyngeal endoderm and optic stalks were greatly reduced in embryos treated with SU5402, S6656, SU5416 and Oxindole I (Figure 6B–6F). However, *dusp6 *expression in the MHB was either completely eliminated by SU5402 or only mildly affected by the other compounds in treated embryos (Figure 6B–6F). These results suggest that the MHB represents a region of highest levels of FGF activity in the embryo during these developmental stages. Finally, compounds that did not alter overall d2EGFP fluorescence (SU11652 and SU4312) in treated FGF reporter line also did not exhibit changes in *dusp6 *transcript levels (Figure 6G &6H).

### Specificity of small molecule FGF signalling inhibitors can be evaluated with *Tg(Fli:EGFP)*^*y*1 ^transgenic embryos

Since the results obtained with the VEGFR inhibitors, SU11652, SU1433 and SU4312, were predominantly negative and did not alter d2EGFP expression in the FGF reporter embryos, we wanted to confirm that these compounds were permeable in the embryo and have an effect on zebrafish VEGR signalling. One approach is to test the activity of these compounds in a zebrafish angiogenesis assay. Such assays have been developed to screen for novel antiangiogenic compounds that can block the formation of the zebrafish intersegmental vessels (ISV), and transgenic lines that delineate the ISV have been used for such assays [35,64]. For our studies we analysed ISV outgrowth in the *Tg(Fli1:EGFP)*^*y*1 ^line that expresses eGFP under the control of the *Fli1 *promoter [65]. To determine the effects of these VEGFR inhibitors on ISV outgrowth, we treated *Tg(Fli1:EGFP)*^*y*1^transgenic embryos at 24 hpf for 8 hours. Treatment within this limited temporal window would reveal whether a compound could specifically block VEGFR signalling and ISV outgrowth as somites, dorsal aorta and posterior cardinal vein would have already been established at 24 hpf. Furthermore previous studies have highlighted the requirement for active VEGFR signalling that is relayed through PLCγ and AKT to form the ISV using a similar time frame [64-66].

**Figure 7:**
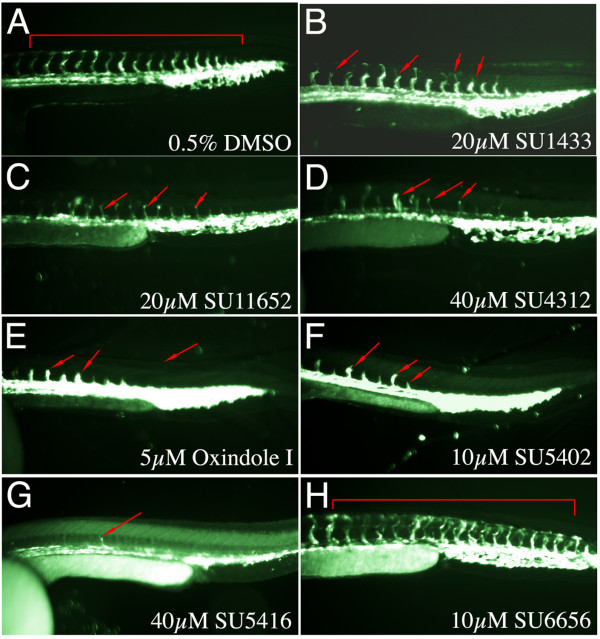
**Specificity of Indolinones towards FGFR versus VEGFR signalling**. **(A-H) **Lateral trunk views of 32 hpf *Tg(Fli1:EGFP)*^*y*1 ^embryos treated with the chemicals shown on the bottom left. **(A) **DMSO control shows embryo with normal expression of EGFP within the intersegmental vessels. **(B) **20 μM SU1433, **(C) **SU11652, **(D) **SU4312 **(E) **Oxindole I, **(F) **SU5402, and **(G) **SU5416 all suppressed ISV outgrowth as indicated by red arrows. **(H) **SU6656 in contrast did not alter ISV formation.

We first treated *Tg(Fli1:EGFP)*^*y*1 ^embryos with SU1433, SU11652 and SU4312, compounds that did not alter fluorescence in the FGF reporter embryos, to determine if these compounds can block VEGFR signalling in the zebrafish. As predicted, all three compounds suppressed ISV outgrowth in the transgenic embryos, thus exhibiting specificity towards VEGFRs, and confirming that these compounds are permeable in the embryo (Figure 7B–D). Similar effects on ISV outgrowth were noted with Oxindole I, a compound that blocked FGF signalling, however at a much lower dose (5 μM) that was required to block fluorescence in *Tg(Dusp6:d2EGFP)*^*pt*6 ^embryos (Figure 7E). This result suggests that Oxindole I has a higher activity towards VEGFR signalling. We next assayed the effects of the FGF receptor inhibitor, SU5402, on ISV outgrowth. SU5402 treated *Tg(Fli1:EGFP)*^*y*1 ^embryos also resulted in the suppression of ISV formation (Figure 7F). The original description of SU5402 had provided evidence that this compound would interact with the ATP binding domain of the VEGFR and was supported by unpublished data that SU5402 did block VEGFR signalling in tissue culture experiments, yet SU5402 is often used as a specific FGFR inhibitor. Thus we stress that the effects of SU5402 treatment may not exclusively reflect a blockade of FGFRs.

SU4312 and SU5416, two compounds that have previously been shown to inhibit VEGF signalling also inhibited ISV outgrowth (Figure 7F &7G) [51,54,67]. Thus SU5416 strongly suppressed both VEGF and FGF signalling in our assays, while SU4312 was specific for VEGF signalling. In contrast, treatment of *Tg(Fli:EGFP)*^*y*1 ^embryos with the inhibitor of Src kinases, SU6656, did not block ISV outgrowth, suggesting that VEGF signalling does not require Src kinases to direct vessel formation (Figure 7H).

**Figure 8:**
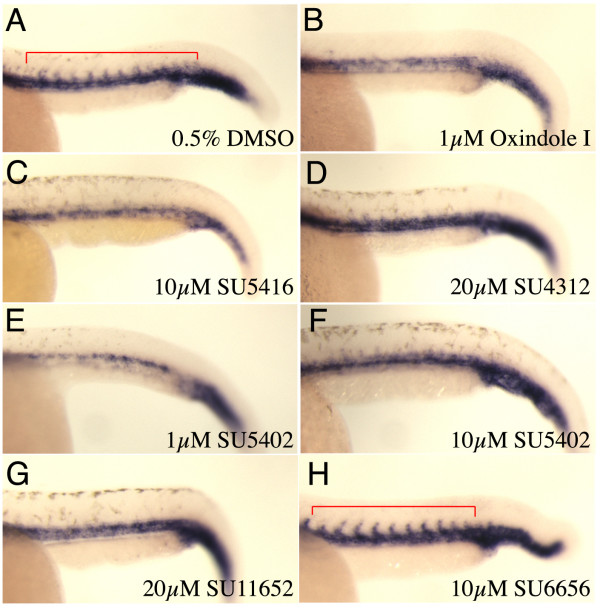
**Expression of *fli1 *in intersegmental vessels in indolinone treated embryos**. **(A-H) **Lateral trunk views of *fli mRNA *expression within the ISV. **(A) **DMSO control shows vessel sprouts at 28 hpf. **(B-G) **Embryos treated with the compounds indicated show loss of ISV sprouts, while in **(H) **SU6656 did not affect these vessels.

To confirm our findings that VEGFR inhibitors did indeed block intersegmental vessel outgrowth, we performed *in situ *hybridisation studies to detect the presence of these vessels through *fli1 *expression. Treatment of embryos with 1 μM Oxindole 1, 10 μM SU5416, 20 μM SU4312, 1 μM SU5402, or with 20 μM SU11652 prevented ISV formation as determined by loss of *fli1 *expression in the intersegmental sprouts (Figure 8B–G). Again these results confirm that SU5402 can block VEGFR signalling in the zebrafish and is not a specific inhibitor of FGFRs. In contrast, expression of *fli1 *within the ISV was unaffected in embryos treated with 10 μM SU6656, confirming the notion that Src kinases are not required in ISV outgrowth (Figure 8H).

## Discussion

In this paper we present the generation of a zebrafish transgenic line that reports on FGF signalling during embryonic development. Expression of d2EGFP in these embryos is regulated by FGF signalling and maps to domains where activated ERKs have been detected [7-12]. Time-lapse imaging of transgenic embryos shows the dynamic expression of d2EGFP throughout embryogenesis. These movies demarcate the formation of Kupffer's vesicle, as d2EGFP labels the DFCs from gastrulation onwards. Since Kupffer's vesicle plays a critical role in the determination of left-right polarity these lines will provide a valuable tool to access the integrity of Kupffer's vesicle formation in left-right polarity mutants [41,42]. Furthermore, these lines provides a way to identify DFCs in post-gastrulation but pre-Kupffer's vesicle stages in live embryos. To our knowledge this is the only line that specifically labels the DFCs with fluorescence as they migrate during gastrulation to eventually form Kupffer's vesicle. This is in contrast to the *Tg(twhh:GFP) *line that also expresses GFP in Kupffer's vesicle, as GFP expression was only detected once the vesicle had formed at the 6-somite stage [68].

While a majority of the d2EGFP expression resembles that of *dusp6 mRNA *during development, a notable exception is the lack of d2EGFP expression within the developing somites and tail bud, two domains where FGFs and Erk activation have been described [3,12,31]. Since only 10 Kb of upstream promoter sequence was used in generating the reporter construct, it is likely that the somitic and tail bud enhancers elements are not part of this sequence.

The expression of d2EGFP is under the control of FGF signalling as manipulation of FGF activity in transgenic embryos resulted in altered d2EGFP expression. These lines will provide useful tools to analyze the integrity of FGF signalling under experimental conditions such as antisense MOs injections or in mutant strains. Previously, Balciunas *et al*. described a transposon-mediated enhancer trapped line that contains an EGFP reporter inserted 30 Kb within the *Dusp6 *locus (ET7 line) [69]. In the ET7 transgenic embryos, EGFP is detected within the MHB, pharyngeal endoderm and somites at 24 hpf, suggesting that *Dusp6 *enhancers may regulate the expression of eGFP [69]. However, it has not been demonstrated if EGFP expression in the ET7 embryos can respond to experimental changes in FGF signalling [69]. We believe that *Tg(Dusp6:d2EGFP)*^*pt*6 ^represent the first description of vertebrate transgenic embryos that can report on FGF activity *in vivo*.

We have used these transgenic lines to validate an approach to screen for small molecules that can modulate FGF signalling. Our results show that within 6 hours of treatment with the FGFR inhibitor, SU5402, complete suppression of de2GFP expression ensued, confirming that these lines can rapidly identify compounds that modulate FGF signal transduction in the embryo. Since FGFRs are members of a larger family of receptor tyrosine kinases we wanted to determine if compounds that are structurally related to SU5402 could also block FGF signalling in the embryo. We determined the specificity of several VEGFR and PDGFR inhibitors that contain the same indolinone backbone as SU5402. From our studies it was clear that SU5416 and Oxindole I, two inhibitors of VEGFR could block d2EGFP expression in the FGF reporter embryos. Furthermore, from these studies we also revealed a putative role for Src kinases in relaying FGF signals in the zebrafish as treatment with Src kinase inhibitors, PP2 and SU6656, suppressed d2EGFP fluorescence. Since several members of this kinase family are expressed early and within cells that receive FGF signals in the developing embryo, including Yes and Fyn, the preferred targets of SU6656, it is reasonable to think that these Kinases are involved in FGF signalling [70-72].

We have coupled the pilot screen with the *Tg(Fli1:EGFP)*^*y*1 ^transgenic line to assay the specificity of these compounds between FGFR and VEGFR signalling. We confirm that SU5416, SU4312, SU11652, Oxindole I and surprisingly SU5402 exhibited inhibitory effects on ISV outgrowth and probably a result of VEGFR inhibition. In the original study, a crystal structure of SU5402 complexed with the FGFR1 kinase domain highlighted the exact peptide residues that interfaced with the compound [48]. Since these residues are also highly conserved with the VEGFRs kinase domain, it was postulated that SU5402 could interact with the VEGFRs [48]. Furthermore the authors refer to unpublished observations that SU5402 did inhibit VEGFR signalling in living cells [48]. Our studies reveal that SU5402 can block ISV outgrowth, which we interpret as a result of VEGFR inhibition in the zebrafish embryo. Alternatively, our data could imply that FGFRs are required for ISV outgrowths given that FGFs are known to play a role in angiogenesis [53]. However from the published literature, FGFR1-4 are not expressed in the ISV to support the role for this pathway in vasculogenesis [25,26]. SU5402 has been used extensively as a FGFR inhibitor in many studies. In light of these findings, other experiments should be considered to test whether SU5402 might also block VEGFR signalling to elicit the observed phenotypes.

## Conclusion

This study describes the generation of transgenic zebrafish, *Tg(Dusp6:d2EGFP)*^*pt*6^, that reports on FGF activity during development. The expression of d2EGFP mirrors the expression of several FGF ligands in the early embryo and will provide a tool to analyse FGF signalling under various experimental conditions. We have performed a pilot screen to validate these lines in chemical screens to identify novel compounds that can modulate FGF activity. This rapid screening protocol can be coupled with the *Tg(Fli1:EGFP)*^*y*1 ^line to eliminate compounds that can potentially cross react with the VEGFR pathway. Finally, acridine orange staining will further eliminate toxic compounds, thus by following these procedures it is possible to identify chemicals that specifically modulate FGF signalling *in vivo*.

## Methods

### Generation of *Tg(Dusp6:d2EGFP) *zebrafish

A *Dusp6 *BAC clone was identified by PCR from BAC DNA pools as directed by manufacturers protocols (Genome Systems). A 10 Kb Kpn1 fragment was identified that contain parts of Exon 1 (441 bp) and approximately 9.5 Kb of upstream promoter sequence (see Figure 1A). This Kpn1 fragment was subcloned into the Kpn1 site of pSce1d2EGFP vector (pSce1 vector with d2EGFP cDNA cloned into the multiple cloning site). 20 pg of *pDusp6:d2EGFP *plasmid DNA was injected into the 1-cell embryo with I-Sce 1 (New England Biolabs, Ipswich, MA) restriction enzyme as described in [37]. These Founder F0 injected embryos were raised to adulthood and incrossed to identify transgenic founders. We identified 10 founder lines that expressed d2EGFP within regions of known FGF activity in the developing embryo. Four lines exhibited strong expression throughout development and were maintained. *Tg(Dusp6:d2EGFP)*^*pt*6 ^was used predominantly in this study.

### Zebrafish Microinjection of RNA and antisense Morpholinos

10 pg *fgf8 mRNA*, 20 ng control-MO(5'-CCTCTTACCTCAGTTACAATTTATA-3'), and 10 ng *fgf8-MO *(5'-GAGTCTCATGTTTATAGCCTCAGTA-3') was injected into the 2-cell stage transgenic embryos as described by Tsang *et. al *and Araki *et. al*, respectively [13,73]. Morpholinos were obtained from Gene-tools inc. (Philomath, OR). Embryos were incubated for the desired stage before visualisation under a Leica stereomicroscope and photographed by a Retiga Exi camera (Qimaging, Burnaby, BC Canada). Images were analyzed in Photoshop CS (Adobe, San Jose, CA).

### *In situ *hybridisation

Zebrafish embryos were fixed in 4% paraformaldehyde and whole mount *in situ *hybridisation was preformed with *d2EGFP*, *dusp6 *and *fli1 *RNA probes [11]. *In Situ *methodology as described in Kudoh *et. al*. [14].

### Chemical treatment of transgenic embryos

Five *Tg(Dusp6:d2EGFP)*^*pt*6 ^embryos at 24 hpf were arrayed into individual wells in a 96-well plate. 100 μl of E3, 0.5% DMSO solution was added along with compound at the dose indicated. SU5402 was kindly provided by Pfizer. SU1498, SU11652, AG1433 (SU1433), SU6656, Oxindole I, were all obtained from Calbiochem (EMD biosciences, Inc. San Diego, CA) and SU5416, SU4312 from Sigma-Aldrich (St. Louis, MO). Embryos were analyzed at 6–8 hour post treatment after manual dechorinonation and treatment with tricaine to immobilise for photography. Treated embryos were photographed under the same settings for exposure, gain and magnification for each picture using a MZFLIII (leica) microscrope and fluorescent illumination for GFP using endow cube (Chroma Technology Corp., Rockingham, VT). Qimaging software and the Retiga Exi camera (Qimaging, Burnaby, BC Canada) was used to capture the images. Each experiment was repeated three times to show reproducibility of the assay and at least 4 of the 5 treated embryos exhibited the same phenotype. For treatment of *Tg(Fli:EGFP)*^*y*1 ^embryos, five transgenic embryos were placed into 96-well plates at 24 hpf and incubated with compound until 32 hpf. Treated embryos were manually dechorionated and photographed as described for the FGF reporter assay. Images were analyzed in Adobe Photoshop CS (San Jose, CA) and false coloured under the same parameters.

### Zebrafish imaging

*Tg(Dusp6:d2EGFP)*^*pt*6 ^embryos were placed into a MatTek glass bottom culture dish (MatTek Corp.) at gastrula stage (6 hpf) and held in place with 1% low melting point agarose. Embryos were photographed under low magnification differential contract (DIC) microscopy and fluorescent illumination for GFP using endow cube (Chroma Technology Corp., Rockingham, VT) at 5 min intervals until 24 hpf. Images were analyzed and processed into movies with Metamorph imaging software (Molecular Devices, Dowlingtown PA).

## Authors' contributions

The experiments described in this paper were planned, conducted and analyzed by GAM, SCW and MT as a joint effort. MT isolated the *Dusp6 *promoter, generated the transgenic lines, described the expression of d2EGFP, and performed experiments to determine that these lines are responsive to FGFs. MT and GAM conceived the chemical screen test and GAM performed the experiments detailed in the pilot chemical screen and the *in situ *hybridisation. SCW was responsible for the time-lapse imaging of these lines. MT drafted the manuscript and all authors read and approved the final version.

## Supplementary Material

Additional file 1 Movie showing d2EGFP expression in Dorsal Forerunner Cells (DFCs). Movie of developing *Tg(Dusp6:d2EGFP)*^*pt*6 ^embryo from late gastrula stage until bud stage. The movie shows a group of fluorescent cells, the DFCs as they coalesce towards the caudal region of the embryo. Click here for file

Additional file 2 Movie showing d2EGFP expression in the developing embryo. Time-laspe imaging of developing *Tg(Dusp6:d2EGFP)*^*pt*6 ^embryo from gastrula stage until 24-somite stage. Movie highlights the dynamic expression of d2EGFP within the developing embryo. The movie of the fluorescent embryo was overlayed onto the DIC-imaged movie to show clearly the developing embryo. Note expression of d2EGFP within the developing hindbrain, MHB and Kupffer's vesicle. Click here for file

Additional file 3 Movie showing d2EGFP expression in Kupffer's vesicle. Time-laspe imaging of *Tg(Dusp6:d2EGFP)*^*pt*6 ^embryo from gastrula stage until 24-somite stage. GFP movie showing the formation of Kupffer's vesicle from the 6-somite stage right until after this structure collapses at the 20-somite stage. Note Kupffer's vesicle cells collapse inwards and begin to migrate towards the tail bud. Click here for file

Additional file 4 Movie showing d2EGFP expression in Kupffer's vesicle with DIC overlay. This is the same movie as Supplemental Movie 3, but overlayed with the DIC-imaged movie to show structures of the developing embryo. Click here for file

Additional file 5 Diagram of the small molecules used in this study. The majority of the chemicals used in the pilot screen are related in structure and contain the indolinone backbone as described for SU5402. Click here for file
