# Supplementary figures and images for: Generation of FGF reporter transgenic zebrafish and their utility in chemical screens
Source: BMC Dev Biol. 2007 Jun 6;7:62. doi: 10.1186/1471-213X-7-62 (PMC1904198; doi:10.1186/1471-213X-7-62)

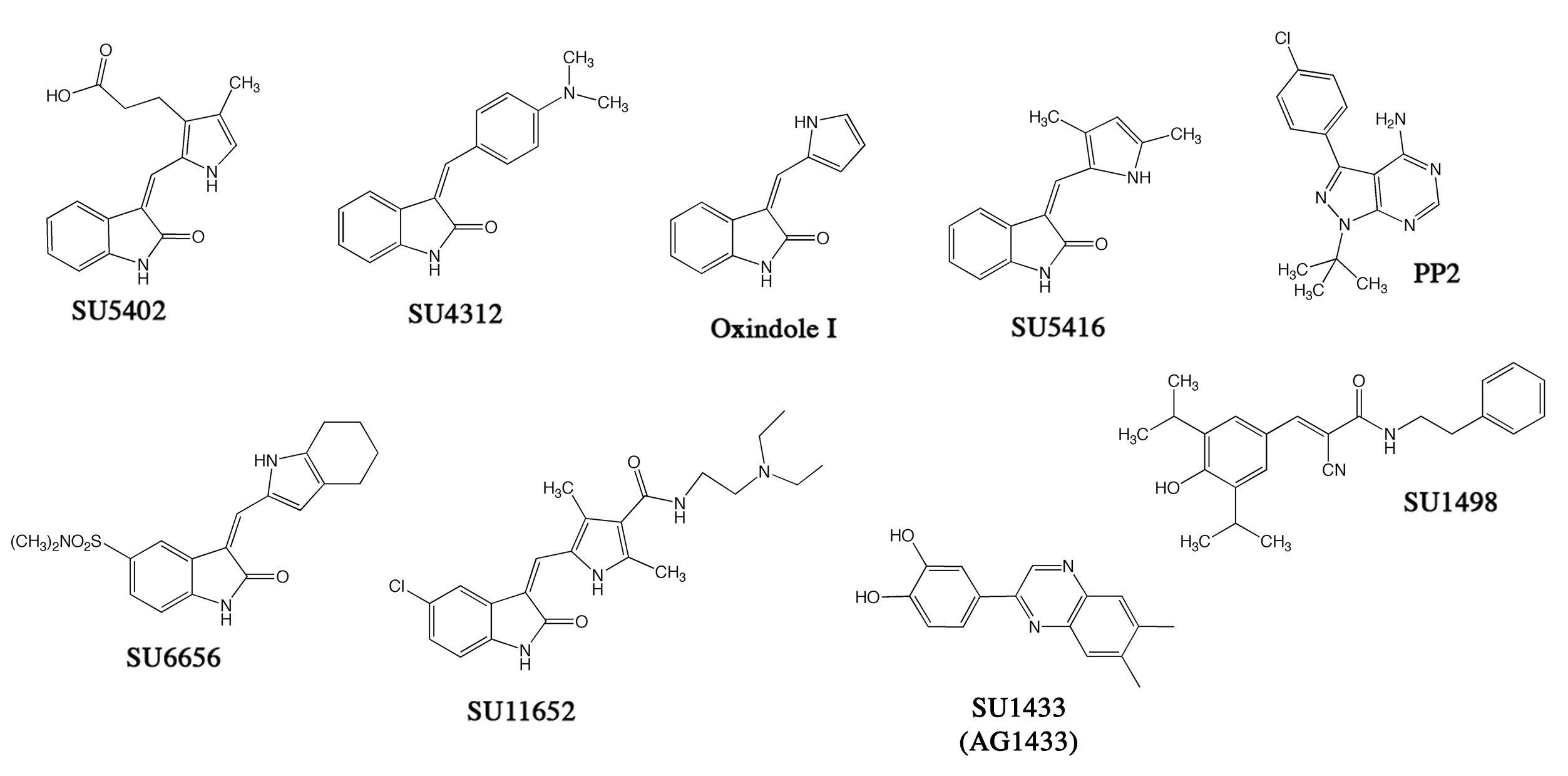

Supplement: Additional file 5 — Diagram of the small molecules used in this study. The majority of the chemicals used in the pilot screen are related in structure and contain the indolinone backbone as described for SU5402. [file 1471-213X-7-62-S5.png]
